# Combining A4R and MCDA in priority setting for health

**DOI:** 10.1186/s12962-018-0124-9

**Published:** 2018-11-09

**Authors:** Norman Daniels

**Affiliations:** Harvard University, Boston, MA USA

**Keywords:** Multi criteria decision analysis, Accountability for reasonableness, Legitimacy, Stakeholders

## Abstract

Multiple criteria decision analysis (MCDA) has been proposed as a method for determining the criteria to be used in health technology assessment. A standard criticism of MCDA is that it lacks attention to securing legitimacy for its decisions. The relevance condition of A4R has been criticized for its vagueness because it lacks a focus on criteria selection. Combining the two methods addresses the central criticisms of each and provides a way of addressing the problem of priority setting for health.

Every health system must set priorities for health care because each has a limited health-care budget. Even systems that do not have a global budget must be sustainable. Setting priorities for health means getting agreement on the criteria to be used for adding new technologies to a health system. Multiple criteria decision analysis (MCDA) [1, 2] has been proposed as a method for determining the criteria to be used in health technology assessment (HTA). But, a standard criticism of MCDA is that it lacks attention to securing legitimacy for its decisions. Accountability for reasonableness (A4R) [3] proposes four conditions (publicity, relevance, revisability and enforcement) that must be met if legitimacy and fairness are to be ascribed to decisions about priority setting. The relevance condition has been criticized for its vagueness because it lacks a focus on criteria selection.

Combining the two methods addresses the central criticisms of each and provides a way of addressing the problem of priority setting for health. As a developer of the A4R approach to enhancing the legitimacy and fairness of decision making, I endorse the combination, as does Rob Baltussen (a major figure in the development of MCDA). Mireille Goetghebeur, President of EVIDEM, has played a prominent role in promoting the combination of the two, transforming the approach to priority setting. It is possible for the selection of criteria to be based on the democratic deliberation of a broad range of stakeholders in the decision, and that provides a clear way to combine MCDA with A4R.

There remain several areas of research that will develop this proposal. One of the research items that must be addressed is managing the deliberation effectively. While vested interests, e.g. among pharmaceutical companies, must be included among stakeholders, they cannot be allowed to dominate the process. We also know that we do not want irrelevant traits of stakeholders, such as the charisma they bring or their showmanship in presentation, to bias findings, but we need evidence about how to correct for these influences. Similarly, we need to do research on the selection of stakeholders and their importance at the different levels at which decisions are made in a health system: which stakeholders are important to include at what levels of decision making is key to know. We also must do better research of the outcomes of compliance with the conditions required by A4R. Research results must be incorporated in stakeholder training.

Other key issues include how we measure legitimacy and how we measure the fairness of decision making. We might be attracted to survey methods for assessing the enhancement of legitimacy. This approach may be the main way to assess improvements in the legitimacy of a combined approach, of using A4R and MCDA, might have. But should we assess any improvements in the fairness of decisions? The problem arises because we may lack agreement on a substantive notion of fairness, and that may be the basis for resorting to a fair process. What should we say if more people think there is greater fairness in a decision, but they couple this conclusion with racist or misogynist views?

## Conclusion

We should combine MCDA and A4R to address the main criticisms of each, but research is needed on how to manage the process of priority setting and on how to measure increased legitimacy and fairness.
